# Novel Histopathological and Molecular Effects of Natural Compound Pellitorine on Larval Midgut Epithelium and Anal Gills of *Aedes aegypti*

**DOI:** 10.1371/journal.pone.0080226

**Published:** 2013-11-18

**Authors:** Haribalan Perumalsamy, Jun-Ran Kim, Sang Mi Oh, Je Won Jung, Young-Joon Ahn, Hyung Wook Kwon

**Affiliations:** 1 Research Institute for Agriculture and Life Science, Seoul National University, Seoul, Republic of Korea; 2 WCU Biomodulation Major, Department of Agricultural Biotechnology, Seoul National University, Seoul, Republic of Korea; Johns Hopkins University, Bloomberg School of Public Health, United States of America

## Abstract

The yellow fever mosquito, *Aedes aegypti*, is a vector for transmitting dengue fever and yellow fever. In this study, we assessed the histopathological and molecular effects of pellitorine, an isobutylamide alkaloid, on the third instar of *Ae. aegypti* larvae. At 5 mg/l concentration of pellitorine, the whole body of the treated larvae became dark in color, particularly damaged thorax and abdominal regions. Pellitorine was targeted mainly on midgut epithelium and anal gills, indicating variably dramatic degenerative responses of the midgut through a sequential epithelial disorganization. The anterior and posterior midgut was entirely necrosed, bearing only gut lumen residues inside the peritrophic membranes. Pellitorine caused comprehensive damage of anal gill cells and branches of tracheole and debris was found in hemolymph of the anal gills. RT-PCR analysis indicates that the compound inhibited gene expression encoding V-type H^+^-ATPase and aquaporine 4 after treatment with 2.21 mg/l pellitorine. These results verify that pellitorine merits further study as a potential larvicide with a specific target site and a lead molecule for the control of mosquito populations.

## Introduction

The yellow fever mosquito, *Aedes aegypti*, is cosmopolitan, abundant and a vector for transmitting several human diseases such as dengue fever, Chikungunya, and yellow fever [1]. More than 2.5 billion people are at risk of dengue infection over in 100 countries worldwide. There may be 50∼100 million dengue infections every year, including 22,000 deaths annually, mostly among children [2]. A number of mosquitoes are distinctly increasing in incidence with a high occurrence of dengue fever worldwide due to global warming, increased international travel, and tainted fresh water pools [1], [3]. It is extremely difficult to control *Ae. aegypti* because they adapt well to the environment with high resilience or with the ability to rapidly bounce back to initial numbers after disturbances resulting from natural phenomena or human interventions [4]. In addition, a serious problem with the mosquito species is their ability to rapidly evolve resistance to conventional insecticides such as acetylcholinesterase (AChE) inhibitors, axonic nerve poisons such as pyrethroids, and insect growth regulators [5]. Therefore, there is a critical need for the development of selective control alternatives with novel target sites in mosquitoes.

**Figure 1:**
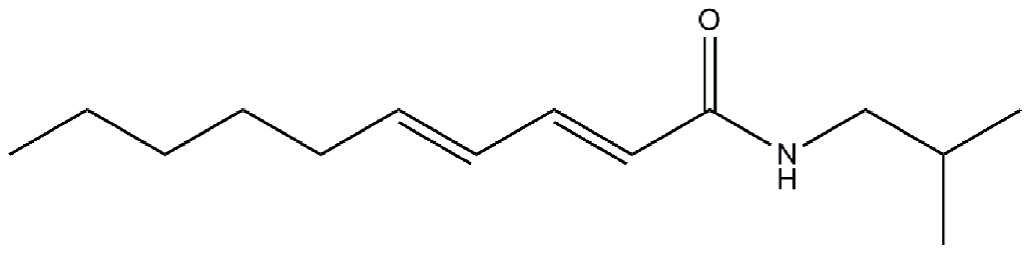
Structure of the isobutylamide alkaloid pellitorine.

Plant secondary metabolites (PSMs) have been suggested as alternative sources for conventional biocides [6]–[8]. This approach is appealing largely because they constitute a potential source of bioactive chemicals that have been perceived by the general public as relatively safe and with fewer risks to the environment, and with minimal impacts to human and animal health [6]–[8]. Unlike conventional insecticides, certain PSMs can act at multiple and novel target sites [9]–[11], thereby reducing the potential for resistance [12], [13]. Histopathological studies revealed that the midgut of insects is one of the main target organs for many xenobiotics, including PSMs [14]–[16] and bacterial endotoxins (*Bacillus thuringiensis* and *Bacillus sphaericus*) [17], [18]. In particular, it was initially reported that the isobutylamide alkaloid pellitorine (Fig. 1) had potent larvicidal activity against third instar *Ae. aegypti* larvae [12]. However, no information is available concerning the histopathological effects of natural pellitorine on *Ae. aegypti* larvae. In our present study, an assessment is made of the histopathological alterations in midgut epithelial cells and anal gills in the third instar larvae of *Ae. aegypti* following exposure to pellitorine using a fluorescent microscopy, a confocal laser scanning microscopy, and a transmission electron microscopy.

In order to deal with these insults to hemolymph homeostasis, larval and adult mosquitoes rapidly respond and restore water and ion balance. Four anal gills surrounding the anal opening are the primary sites of Na+ and Cl– absorption in mosquito larva with which ion and water regulation in hemolymph remains stable [19]. The osmotic uptake of water at the anal gills is the primary external site of ion uptake, normally contributing to 33% of body weight gain per day [20]–[22]. It is believed that the presence of aquaporins (AQPs), especially Aquaporin 4 (AaAQP4) which acts as water channels, may facilitate the movement of water across these tissues [23]. In addition, the anal gills of larval *A. aegypti* serve as the major site for Na^+^, Cl^–^ and K^+^ uptake by H^+^-ATPase and Na^+^/K^+^-ATPase [22], [24].

In this study, we have observed the gene expression analysis of both V-type-H+-ATPase and aquaporin 4 (AaAQP4) in anal gills after treatment with pellitorine was employed to investigate a possible target site of the alkaloid.

## Materials and Methods

### Chemicals and Reagents

Pellitorine was obtained from the root of *Asarum heterotropoides* as reported previously [12]. Triton X-100 was obtained from Shinyo Pure Chemicals (Osaka, Japan). All of the other chemicals and reagents used in this study were of reagent-grade quality and available commercially.

### Mosquitoes

The stock cultures of the insecticide-susceptible *Ae. aegypti* were maintained in the laboratory without exposure to any known insecticide [25]. Larvae were reared in plastic trays (24×35×5 cm) containing 0.5 g of sterilized diet (40-mesh chick chow powder/yeast, 1/1 by weight). Adults were maintained on a 10% sucrose solution and blood fed on live mice. All stages were held at 27±1°C and 65–75% relative humidity under a 16∶8 h light∶dark cycle.

### Treatment with Natural Pellitorine

Natural pellitorine was used for treatment of third instar *Ae. aegypti* larvae during histopathological testing. A 5 mg/l quantity of the compound in methanol was suspended in distilled water with Triton X-100 (20 µl/l), which is equivalent to approximately twofold quantities of the LC_50_ value (2.21 mg/l) of the compound [12]. For gene expression level observation we have used LC_50_ value (2.21 mg/l), because the mosquito larvae should be active rather than having paralysis effect. Groups of 20 mosquito larvae were put into paper cups (270 ml) containing the test solution (250 ml). Controls received methanol–Triton X-100 solution in distilled water.

Treated and control (methanol–Triton X-100 solution only) larvae were held under the same conditions as those used for colony maintenance for 24 h. Larvae were considered dead if its body and appendages did not move when it was prodded with a fine wooden dowel. All treatments were replicated three times using 20 larvae per replicate.

### Light Microscopic Analysis

The pellitorine-treated and -untreated (control) third instar *Ae. aegypti* larvae were put on microscope slides at room temperature for light microscopy. Morphological observations were made with a Leica EZ4 HD equipped with an Integrated 3.0 Mega-Pixel CMOS camera (Heerbrugg, Switzerland).

### Histological Analysis by Cason's Trichome Staining

The treated and control third instar *Ae. aegypti* larvae were immediately fixed in 4% paraformaldehyde (PFA) buffer solution (pH, 7.4) at 4°C overnight. Paraffin-embedded preparations of the larvae were sectioned at 10 µm thickness by using a Thermo Scientific Microm HM 340E rotary microcotome (Walldorf, Germany). Sections were dried at 40°C overnight and subsequently dewaxed with Fisher Scientific CitriSolv (Fair Lawn, NJ) and rehydrated with a series of ethanol to phosphate-buffered saline (PBS) solution as described previously [26]. Triple color staining was carried out using Cason's trichrome staining procedures [27]. In brief, rehydrated paraffin sections were soaked into Cason's trichrome stain for 15 min, and slides were gently swashed in tap water and subsequently distilled water three times. Excess of water was removed and samples were mounted with EMS permount (Hatfield, PA). Images were observed and captured using an Olympus BX43 fluorescent microscope (Tokyo, Japan).

### Immunostaining Analysis

Rehydrated samples mentioned previously were subsequently subjected to immunostaining procedures [26]. To obtain neuron-specific staining, unspecific binding sites in rehydrated samples were blocked for 1 h in 3% Life Technologies normal goat serum (Grand Island, NY) in PBS solution. Afterwards, anti-horseradish peroxidase (HRP) antibody (Jackson ImmunoResearch laboratory, West Grove, PA) conjugated to Alexa Fluor 488 (Jackson ImmunoResearch laboratory) was employed at 1∶250 concentration at 4°C overnight. Samples were washed with PBS solution three times and mounted with Vector Laboratories Vectashield H-1500 mounting medium with DAPI (4′, 6-diamidino-2-phenylindole) (Burlingame, CA). Fluorescent images were captured using a Carl Zeiss LSM 700 confocal laser scanning microscope (Jena, Germany).

### Transmission Electron Microscopic Analysis

The midgut and anal gills of the pellitorine-treated and control larvae were fixed in Karnovsky's fixative 2% (v/v) glutaraldehyde and 2% (v/v) PFA in 0.05 M sodium cacodylate buffer, pH 7.2 at 4°C in darkness for 2–4 h, and washed with the same buffer three times [28]. The specimens were postfixed with 1% (w/v) osmium tetroxide in the same buffer at 4°C for 2 h, and washed with distilled water three times. The postfixed specimens were dehydrated through a graded series of ethanol increasing concentrations up to 100% for 15 min. The specimens were further treated with propylene oxide two times each for 15 min as a transitional fluid, and embedded in Spurr's resin [29]. Ultrathin sections (approximately 50 nm thickness) were cut with a RMC MT-X ultramicrotome (Tucson, AZ), stained with 2% aqueous uranyl acetate for 7 min at room temperature and with Reynolds lead citrate [30] for 7 min. The sections were mounted on copper grids, and the micrographs were obtained from a Carl Zeiss Libra 120 Plus transmission electron microscope (TEM) (Jena, Germany) at 80 kV. Observations were taken of 20 larvae under the TEM.

### Analysis of Gene Expression

Total RNAs were isolated from the anal gills of fifty larvae using a Qiagen RNeasy Mini Kit (Valencia, CA). Using 1 µg of total RNA, cDNA was synthesized with oligo-dT with Invitrogen Superscript III enzyme (Grand Island, NY). Then, using a template of 1 µl of synthesized cDNA, polymerase chain reaction (PCR) amplification was performed with gene specific primer sets for target genes, AaAQP4 (XM_001647996), and AaV-type-H^+^-ATPase (AF092934). PCR conditions were performed by procedures at 95°C for 5 min, followed by 35 cycles of 95°C for 30 s, 58°C for 30 s, 72°C for 1 min, and a final extension at 72°C for 5 min using an Applied Bioscience Thermal Cycler (Foster City, CA). Aarps7 gene, an *Ae. aegypti* gene, was used as a control for normalization. All experiments were triplicate. The information of primers used to amplify each gene is as follows: Aarps7: forward primer-5′-CTGGAGGATCTGGTCTTC-3′; reverse primer- 5′-GTGTTCAATGGTGGTCTG-3′ (Ref-ID_5572090), AaAQP4: forward primer- 5′-ATGCCACTGCTTGTCCCTAC-3′; reverse primer 5′-TTTCCGAAATGACCTTGGAG-3′ [23], AaV-type H^+^ ATPase: forward primer 5′-GTTGTTCTGGCTCTGCTGTTA-3′; reverse primer- 5′-GAGTGTTCTCGATAAGCCATAATC-3′ [24]. In order to analyze the relative gene expression levels, gene bands on agarose gels were visualized using Bio-Rad Gel Doc XR + Imaging system (Hercules, CA). Subsequently, the band intensity was automatically computed by densitometry standard with Fuji Multi-Gauge version 3.0 software (Tokyo). The relative gene expression levels (%) in treatment groups were calculated as follows: the band intensity of pellitorine-treated group ÷ the band intensity of control group ×100 [31]. Relative gene expression level of control groups was normalized to 100% (Control/Control ×100). Statistical analysis for significant difference in gene expression patterns was tested using Student t-test (SPSS, version 17, USA).

## Results

### Pathological Symptoms by Pellitorine

**Figure 2:**
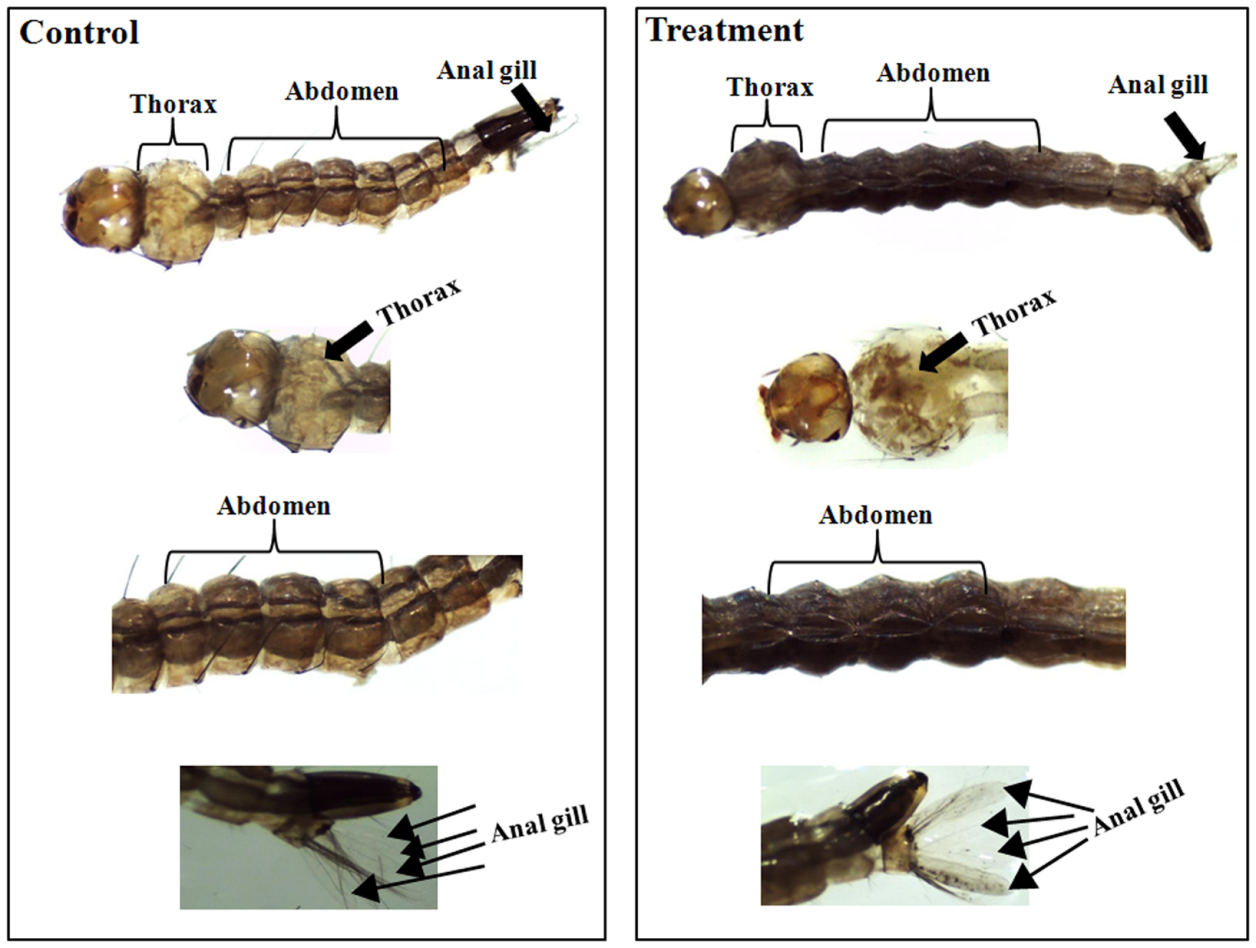
Light micrographs of midgut, thorax, and anal gill parts of third instar *Ae. aegypti* larvae without (A) and with treatment with 5 mg/l of natural pellitorine (B) ×35 magnification. Pellotorine treatment induced toxic effects on many different regions of the body including thorax, abdomen, and anal gills. All experiments were conducted in triplicate in which 20 mosquito larvae were used in each replicate. More than 10 live larvae from control and treated groups were randomly collected and used for histological analysis. All histological observations showed similar results throughout experiments same in other results.

The normal morphology for the whole body of the control third instar *Ae. aegypti* larvae showed the common appearance of the typical structure with well-developed distinguished head, thorax, and abdomen region (Fig. 2A). The whole body of the *Ae. aegypti* larvae treated with pellitorine became dark in color, particularly in the damaged thorax and abdominal region (from 1st to 5th segments). Damaged internal gastric caeca and dark black spots were observed in the thorax region and the anal gills, respectively (Fig. 2B).

### Histopathological Effect of Pellitorine by Cason's Staining

**Figure 3:**
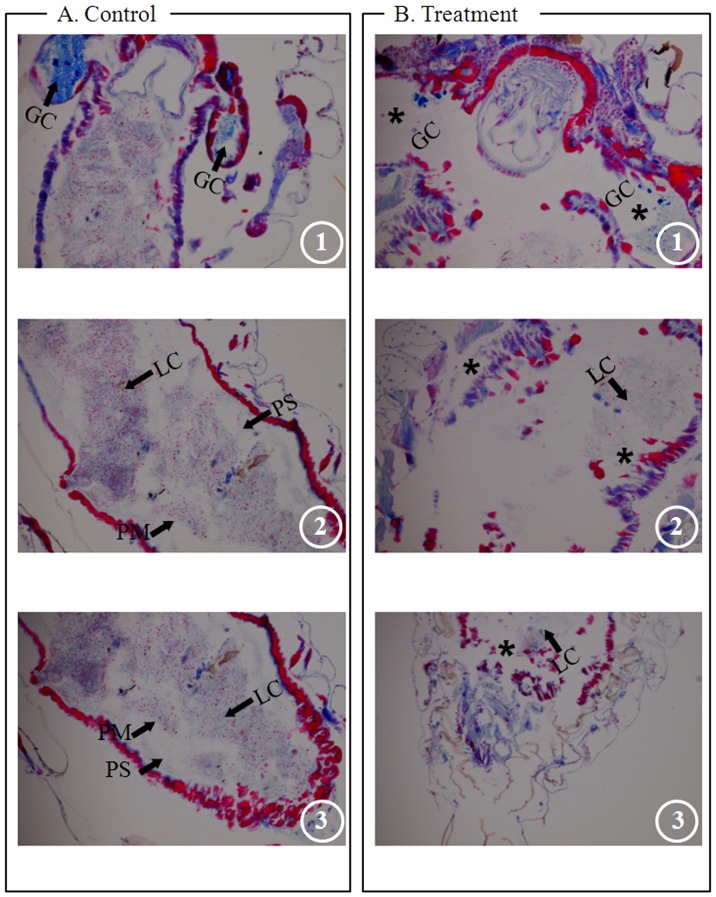
Histology of thorax and midgut regions of third instar *Ae. aegypti* larvae. (A) Control mosquito. A1. anterior midgut region of control larvae has well-developed gastric caeca (GC) and single-layered midgut epithelium. A2. Central midgut region of control larvae consisted of well-developed lumen content (LC), peritrophic space (PS), and peritrophic membrane (PM). A3. Posterior midgut region of control larvae consisted of distinguished midgut epithelial layer, lumen contents, and peritrophic membrane. (B) Treated mosquito with 5 mg/l natural pellitorine showed undistinguished enlarged portion of gastric caeca and damaged single-layered epithelial cells. B1. Pellitorine-treated larvae had an undistinguished enlarged portion of gastric caeca and damaged single-layered epithelial cells (asterisks). B2. Central midgut region of pellitorine-treated larvae showed demolished epithelial layer residues mixed with a few LC (asterisks). B3. Complete damaged residue of epithelial and peritrophic membranes was observed in pellitorine-treated larvae (asterisk).

Well-developed gastric caeca (GC) and single-layered midgut epithelia were observed in the anterior midgut regions of the control *Ae. aegypti* larvae (Fig. 3A1), whereas undistinguished enlarged portions of gastric caeca and damaged single-layered epithelial cells were observed in the pellitorine-treated larvae (Fig. 3B1). The central midgut regions of the control larvae consisted of well-developed lumen contents (LC), and were surrounded by an inner transparent peritrophic membrane (PM) and an outer midgut epithelial layer. Both inner and outer membranes were separated by peritrophic space (PS) (Fig. 3A2). In the central midgut regions of the treated larvae, demolished epithelial layer residues were observed (Fig. 3B2). Distinguished midgut epithelial layers, lumen contents, and peritrophic membranes were observed in the posterior midgut regions of the control larvae (Fig. 3A3), whereas completely damaged residues of epithelial and peritrophic membranes were observed in the pellitorine-treated larvae (Fig. 3B3).

**Figure 4:**
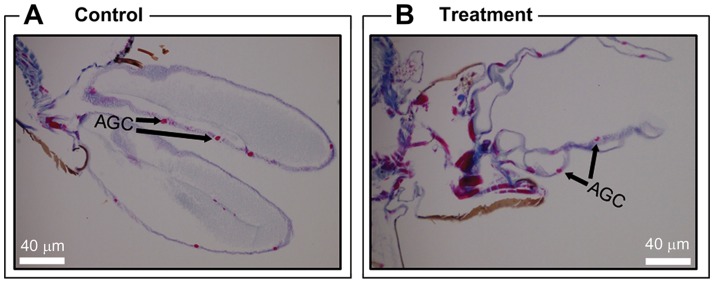
Histology of anal gill region of third instar *Ae. aegypti* larvae. (A) Control larvae showed that the anal gill has inner lined epithelium layer (EP) with well-organized anal gill cells (AGC) surrounded by thick cuticle layer. (B) Undistinguished damaged cuticle leading to completely destroyed anal gill cells and shrinked anal gill of the treated larvae with 5 mg/l natural pellitorine compared to the anal gill of the control larvae.

Well-developed glandular anal gill cells which were connected with rectum through the anal canal were observed in the control *Ae. aegypti* larvae. The anal gills were surrounded by a thick permeable cuticle layer consisted of anal gill cells (Fig. 4A). However, undistinguished damaged cuticle was observed in the pellitorine-treated larvae (Fig. 4B), leading to completely destroyed anal gill cells (Fig. 4B2).

### Histopathological Effect of Pellitorine by Immunostaining

**Figure 5:**
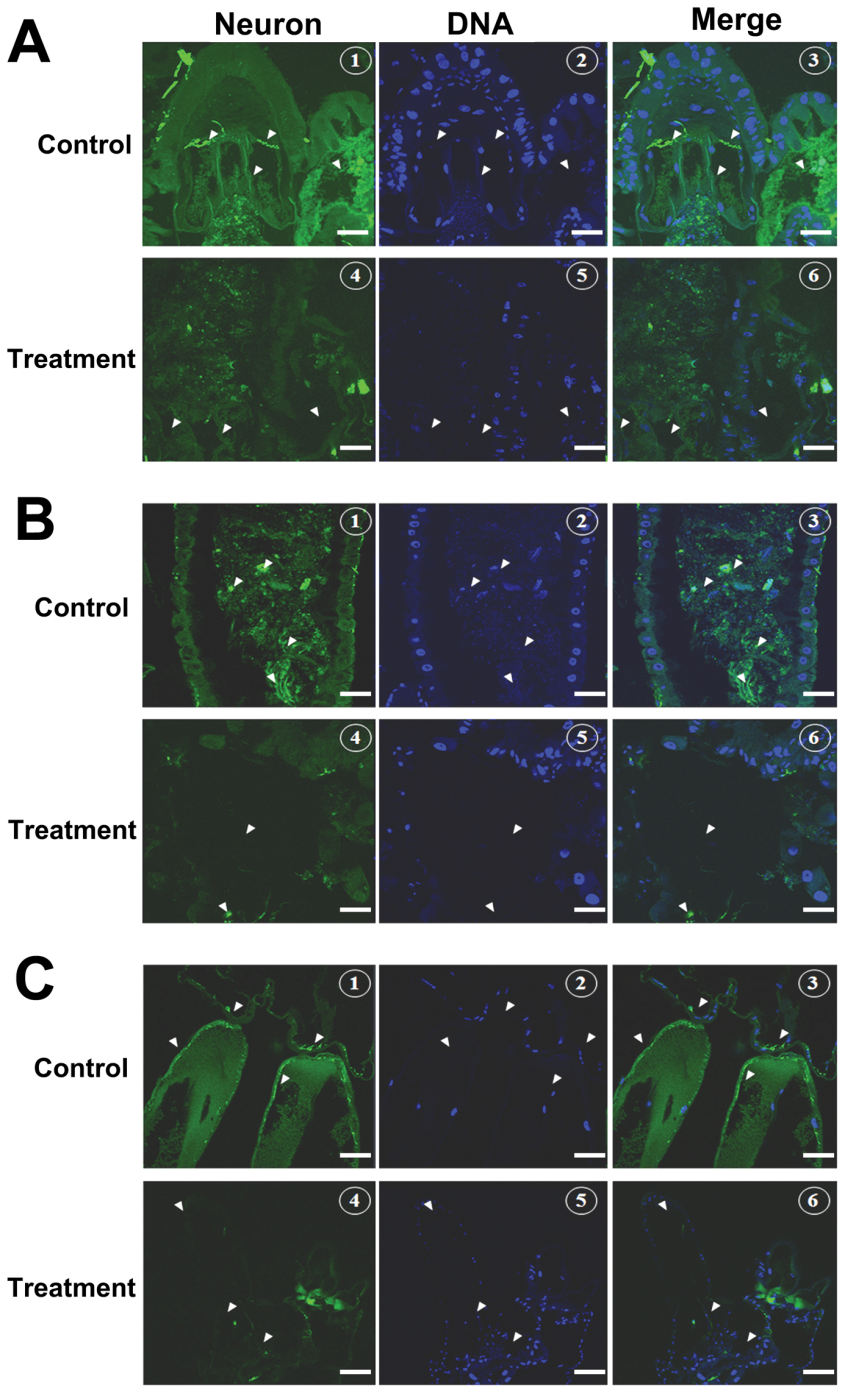
Confocal laser scanning micrograph histology observations by immunostaining. (A1–3) Anterior midgut region of the control larvae showed well-developed cardial nerves and subpopulation of neuron ends in rich neuropils. (A4–6) Cardial nerves in thorax and anterior midgut regions of larvae treated with 5 mg/l alkaloid pellitorine showed disappearance of neural processes. (B1–3) In posterior midgut region, neuronal cells and processes near the epithelial layer of the posterior midgut region were present. (B4–6) Posterior midgut region in the pellitorine-treated larvae also showed the lack of cells in the epithelial layers. (C1–3) Anal gill region of the control larval mosquito showed well-developed neurons elongated throughout large swelling anal gill cells. (C4–6) Neurons were damaged and disappeared in treated larvae. Arrowheads shown in (A) represent cardial nerves, gastric caeca, and neural processes. Arrowheads in (B) represent neuronal cells in epithelial layers of the gut. Arrowheads in (C) indicate neuronal elongation and branches to anal gills. Scale bars depict 40 µm.

Confocal laser scanning micrographs revealed well-developed cardial nerves and subpopulations of neurons ended in rich neuropils in thorax and anterior midgut regions of the control *Ae. aegypti* larvae (Fig. 5A1–3). Particularly in the control larvae, the gastric caeca consisted of well-developed neuropils (Fig. 5A1–3). In contrast, cardial nerves in the thorax and anterior midgut regions of the pellitorine-treated larvae showed disappearance of neuropils, and also in the region of gastric caeca (Fig. 5A4–6). The control larvae contained more neuronal cells and processes around the epithelial layer of the posterior midgut region (Fig. 5B1–3). Few neurons in damaged epithelial layer cells were observed in the posterior midgut regions of the pellitorine-treated larvae (Fig. 5B4–6). Well-developed neurons elongated throughout large swelling anal gill cells were observed in the anal gill regions of the control larvae (Fig. 5C1–3). The anal gill regions of the treated larvae showed damaged and disappearance of neurons (Fig. 5C4–6).

### Histopathological Effect of Pellitorine on Anterior and Posterior Midgut

**Figure 6:**
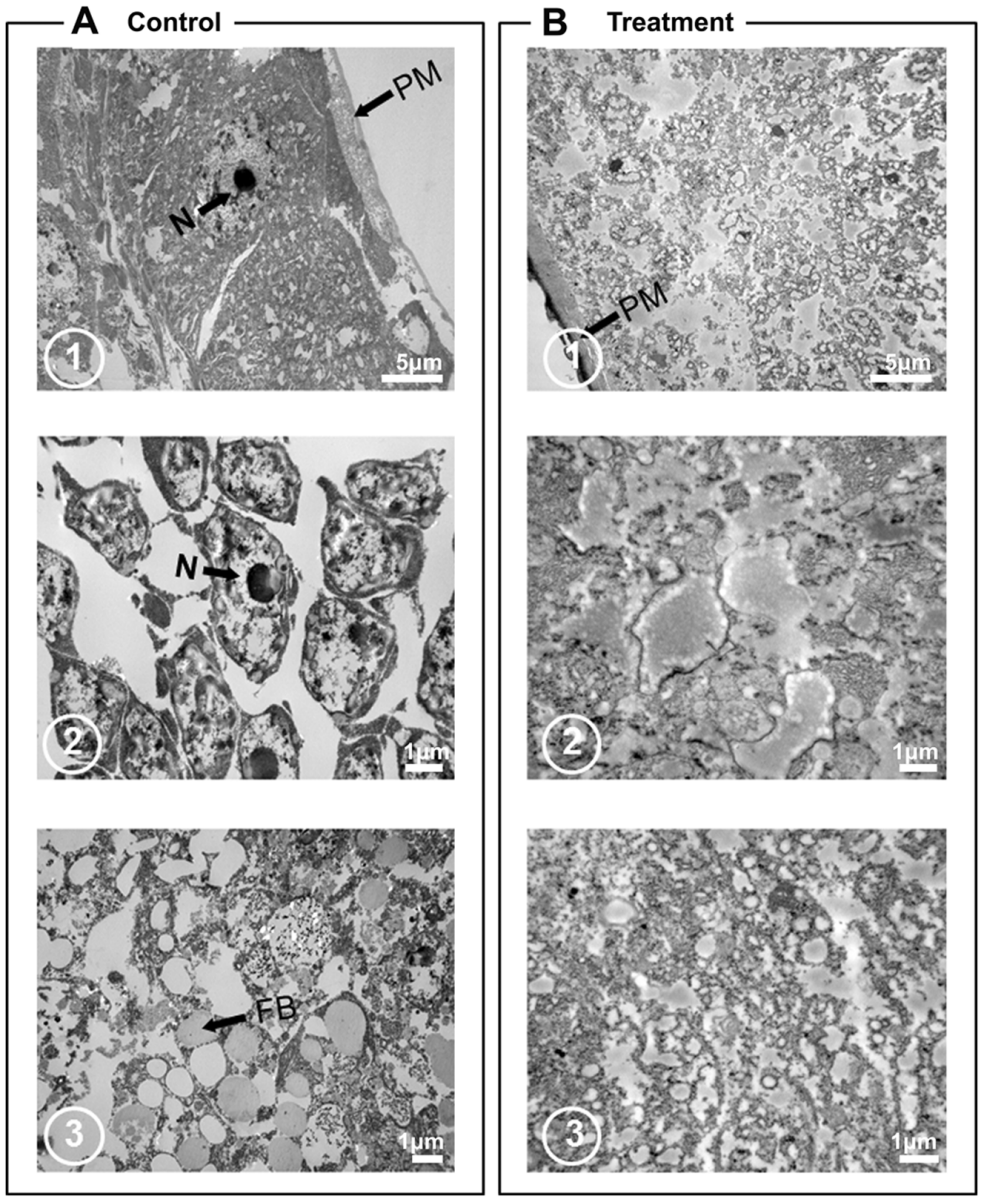
Transmission electronic micrographs of anterior midgut regions of third instar *Ae. aegypti* larvae without (A) and with treatment with 5 mg/l of natural pellitorine (B). A1. A control larva showed well-developed peritrophic membrane (PM) enclosing midgut lumen contents. The midgut lumen cells consist of prominent nucleus and other cellular content present in the cytoplasm. A2. Organization of anterior midgut epithelial cells with large nucleus in a control larva. A3. Anterior midgut lumen content possesses numerous fat body (FB) tissue. B1-2. In the anterior midgut region of larvae treated with 5 mg/l natural pellitorine, all cellular contents including the nucleus were destroyed and cell masses in the cytoplasm were extruded. B3. Fat body tissues of lumen content were demolished by pellitorine treatment. N, nucleus;

A transmission electron (TE) micrographs revealed that a peritrophic membrane (PM) in the anterior midgut regions of the control *Ae. aegypti* larvae enclosed the midgut lumen contents (LC) and is separated from outer surface by midgut epithelia by a narrow peritrophic space (PS) (Fig. 3A2, Fig. 6A1). The anterior midgut regions of the control larvae were composed of well speared cellular contents and also possessed a prominent nucleus (Fig. 6A1). In addition, the anterior midgut epitheial layer consisted of well-developed epithelial cells having a large nucleus (Fig. 6A2). The lumen contents of the anterior midgut regions also contain numerous well-developed fat body tissues (Fig. 6A3).

All of the cellular contents in the anterior midgut regions of the pellitorine-treated larvae were devastated compared to the control larvae. In particular, the nucleus and cells surrounding the nucleus were completely destroyed (Fig 6B1). The anterior midgut epithelial cells showed completely damaged and undistinguished cellular residues (Fig. 6B2). In addition, the fat body tissues in the anterior midgut lumen contents were demolished by pellitorine treatment (Fig. 6B3).

**Figure 7:**
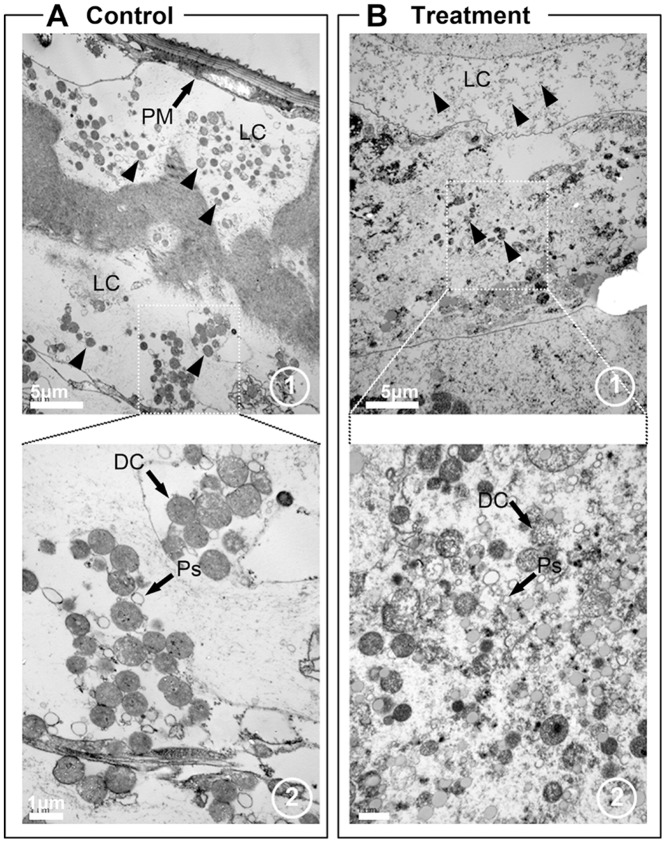
Transmission electronic micrographs of posterior midgut regions of third instar *Ae. aegypti* larvae without (A) and with treatment with 5 mg/l of natural pellitorine (B). A1–2. The posterior midgut region of the control larva was characterized by few epithelial cells with electron-dense cytoplasm. Also, posterior midgut lumen has shown numerous dark cells with nucleus and polysomes. B1. Posterior midgut region of a larva treated with 5 mg/l natural pellitorine showed the compound destroyed all posterior midgut epithelial cells layer and also caused degeneration of dark cells and polysomes. B2. Residual tissues inside the gut lumen were observed in the posterior midgut in a pellitorine-treated larva. PM, peritrophic membrane; LC, lumen contents; PS, polysomes; DC, dark cell

The posterior midgut of the control larvae was characterized by a few epithelial cells (Fig. 3A3) with electron-dense cytoplasm (Figs. 7A1 and A2) as reported previously [15], [32]. The posterior midgut regions showed numerous dark cells within the nucleus and also showed some polysomes in well-developed lumen contents as in control larvae sections (Figs. 7A2). Pellitorine destroyed all posterior midgut epithelial cell layers. In particular, cells in the luminal contents region were completely destroyed (Fig. 7B1) and caused the degeneration of dark cells and polysomes (PS) (Fig. 7B2). An undistinguished cellular layer bearing only residues inside the gut lumen was also observed in the posterior midgut (Fig. 7B2).

### Histopathological Effect of Pellitorine on Anal Gills

**Figure 8:**
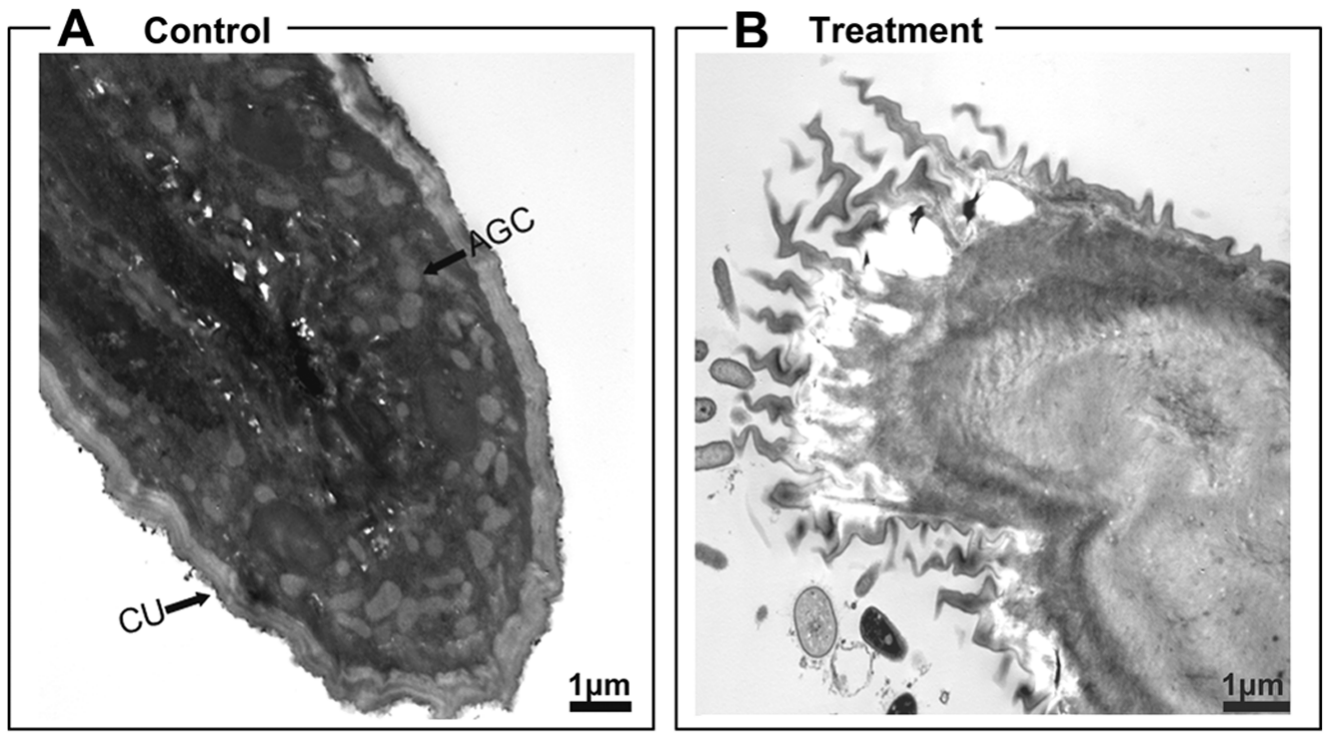
Transmission electronic micrographs of anal gill regions of third instar *Ae. aegypti* larvae. (A) The anal gill of the control larva was surrounded by thick cuticle (CU) and inner surface covered with epithelial layer (EP) having large swelling anal gill cells (AGC) filled with hemolymph. (B) The anal gill of a larva treated with 5 mg/l natural pellitorine showed damaged outer membrane surrounded by a thick cuticle, which led to internal lumen content destruction, particularly degeneration of all anal gill cells.

The TEM study revealed that the anal gill of the control larvae consisted of an epithelia and covered by a thin and relatively permeable cuticle [20], [33]. The epithelium is extensively tracheated with tracheolar anal gill cells, and the anal gill lumen is filled with hemolymph and is continuous with the hemocoel [33] (Fig. 8A). In contrast, the damaged outer membrane was surrounded by a permeable cuticle layer of anal gill in pellitorine-treated larvae, which led to internal cytoplasmic destruction, particularly degeneration of all tracheolar anal gill cells (Fig. 8B).

### Pellitorine-induced Target Gene Expression

**Figure 9:**
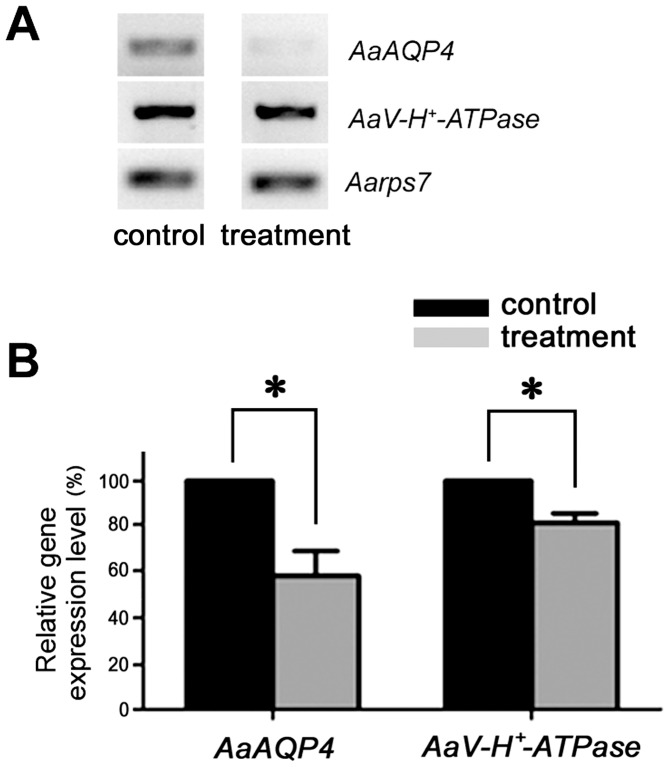
Gene expression patterns by pellitorine treatment. (A) RT-PCR of AaAQP4, AaV-H^+^-ATPase and Aarps7 genes in control and treatment groups. (B) Relative gene expression level of AaV-type H^+^-ATPase and aquaporin 4 (AaAQP4), compared to the expression level of Aarps7 gene. The gene expression of AaV-type H^+^-ATPase and aquaporin 4 (AaAQP4) in the anal gills was inhibited in a larva treated with 5 mg/l natural pellitorine as compared to the control larvae. * indicates significant difference in gene expression levels in control and pellitorine treated groups (Student's *t*-test, N = 3, *P*<0.01).

The transcript expression patterns of V-type H^+^-ATPase that is involved in the Na^+^, Cl^–^, and K^+^ uptake co-transport process and a putative aquaporin protein in anal gills were observed in both control and pellitorine-treated *Ae. aegypti* larvae (Fig. 9). The gene expression of V-type H^+^-ATPase was significantly inhibited in the pellitorine-treated larvae as compared to the control larvae. Similarly, aquaporin protein gene expression level was inhibited in the treated larvae as compared to the control larvae. The expression level of rps7 gene of *Ae. aegypti* (Aarps7) was not affected by both treated and control larvae.

## Discussion

The current microscopic analysis clearly indicates that pellitorine caused histopathological alterations in thorax, midgut, and anal gill regions in the third instar larvae of *Ae. aegypti*. Investigations on the modes of action and the resistance mechanisms of plant-based biocides are of practical importance because they may provide useful information on the most appropriate formulations to be adapted for future commercialization and future resistance management. Also, they may contribute to the development of selective mosquito control alternatives with novel target sites and low toxicity [6], [7]. It has been reported that pellitorine is effective against *Cules pipiens pallens* larvae with high levels of resistance to AChE inhibitors such as chlorpyrifos, fenitrothion, and fenthion as well as axonic nerve poisons such as α-cypermethrin and deltamethrin [12]. These results suggest that the alkaloid pellitorine and the pyrethroid and organophosphate (OP) insecticides do not share a common mode of action. In addition, histopathological investigations indicate that the midgut epithelium is the site of action of plant preparations and PSMs in *Papilio polyxenes* and *Papilio glaucus* [14], some aquatic dipteran larvae [15], [16], and *Schistocerca gregaria* and *Locusta migratoria* [34], *Rhodnius prolixus* [35], and several species of Acridoidea [36]. Midgut epithelium is known to have functions such as ionic and osmotic regulation [32], lipid and carbohydrate storage [32], [37], [38], control of the midgut lumen pH, and the secretion of digestive enzymes and absorption of nutrients [39], [40]. The histopathological effects differ qualitatively according to the localization of organs along the midgut and quantitatively according to the concentration of test material examined, the duration of the treatment, and the taxon [16], [34]. Nasiruddin and Mordue [34] reported that azadirachtin caused some of the initial effects on necrosis, particularly associated with the swelling of the cell and organelles, vesiculation of membranes, and dilation of rough endoplasmic reticulum in locusts. It has been also reported that tannic acid caused dramatic degenerative response of the midgut through a sequential epithelial disorganization in *C. pipiens* larvae [15]. Our current study revealed that pellitorine caused dramatic degenerative responses in thorax and anterior and posterior midgut regions of *Ae. aegypti* larvae by targeting ion transporting cells in gastric caeca of the thorax region and epithelial cells of the anterior and posterior midgut region where osmoregulation-related machineries such as, H+V-ATPase are highly expressed in the anterior midgut of *Ae. aegypti* larvae [24], [41], [42].

The anal gills of mosquito larvae are important primary sites of NaCl uptake, thereby acting to offset the dilution of the hemolymph by the dilute habitat [21], [43], [44]. Donini and O'Donnell [22] confirmed with the use of self-referencing ion-selective microelectrodes that the anal gills of *Ae. aegypti* larvae serve as the major site for Na^+^, Cl^–^, and K^+^ uptake, also complementing the role of the Malpighian tubules and rectum. Additionally, Marusalin et al. [23] proved that putative aquaporin homologs, especially AaAQP4, play an important role in mediating water movement across the anal gill epithelia of the mosquito larvae. However, few information is available with respect to the histopathological as well as gene expression patterns of V-type H^+^-ATPase and AaAQP4 by insecticides or PSMs on anal gills of mosquito larvae. Our present study has revealed that pellitorine inhibited AaAQP4 expression levels. This natural compound may disturb the Na^+^, Cl^–^, and K^+^ co-transport system mainly by the degeneration of anal gill cells and the damage of outer thick permeable cuticle membranes of *Ae. aegypti* larvae. In contrast, the gene expression encoding V-type H^+^-ATPase protein in the Na^+^, Cl^–^, and K^+^ ion co-transport system has shown slight decrease in our study, even though it has shown significant effects on the gene expression level by pellitorine treatment. More detailed examination on ion exchange effects in anal gills by natural pellitorine compound remains to be investigated, even though it has been reported that it is difficult to measure the ion exchange in place due to their morphological characteristics [19]. These original findings indicate that pellitorine caused the histopathological alterations and inhibition of gene expression of V-type H^+^-ATPase and aquaporin protein in the anal gills. The findings may also contribute to a better larvicide mode of action understanding of an alkaloid against *A. aegypti*.

In conclusion, pellitorine caused degenerative responses in the cell organelles of the thorax, midgut regions, and anal gills, possibly by targeting with the osmoregulation system. The alkaloid acts as a potential mosquito larvicide with a specific target site for the control of mosquito populations. Further research is needed to establish the toxicity of aquatic non-target organisms.
